# A Camera-Based Position Correction System for Autonomous Production Line Inspection

**DOI:** 10.3390/s21124071

**Published:** 2021-06-13

**Authors:** Amit Kumar Bedaka, Shao-Chun Lee, Alaa M. Mahmoud, Yong-Sheng Cheng, Chyi-Yeu Lin

**Affiliations:** 1 Department of Mechanical Engineering, National Taiwan University of Science and Technology, Taipei 106, Taiwan; bnbscottlee@gmail.com (S.-C.L.); eng.alaa.mohamed442@gmail.com (A.M.M.); cyongsheng213@gmail.com (Y.-S.C.); 2 Center for Cyber-Physical System Innovation, National Taiwan University of Science and Technology, Taipei 106, Taiwan; 3 Taiwan Building Technology Center, National Taiwan University of Science and Technology, Taipei 106, Taiwan

**Keywords:** automatic optical inspection (AOI), offline programming, position correction, image processing, production line, manipulator, camera-based system

## Abstract

Visual inspection is an important task in manufacturing industries in order to evaluate the completeness and quality of manufactured products. An autonomous robot-guided inspection system was recently developed based on an offline programming (OLP) and RGB-D model system. This system allows a non-expert automatic optical inspection (AOI) engineer to easily perform inspections using scanned data. However, if there is a positioning error due to displacement or rotation of the object, this system cannot be used on a production line. In this study, we developed an automated position correction module to locate an object’s position and correct the robot’s pose and position based on the detected error values in terms of displacement or rotation. The proposed module comprised an automatic hand–eye calibration and the PnP algorithm. The automatic hand–eye calibration was performed using a calibration board to reduce manual error. After calibration, the PnP algorithm calculates the object position error using artificial marker images and compensates for the error to a new object on the production line. The position correction module then automatically maps the defined AOI target positions onto a new object, unless the target position changes. We performed experiments that showed that the robot-guided inspection system with the position correction module effectively performed the desired task. This smart innovative system provides a novel advancement by automating the AOI process on a production line to increase productivity.

## 1. Introduction

The development of industrial automation became quite mature by the 1970s, and many companies have been gradually introducing automated production technology to assist production lines since [1]. The automation of the production line has enabled the manufacturing process to be more efficient, data-based, and unified. Automation technologies have heavily influenced many processes, from loading and unloading objects to sorting, assembly, and packaging [2].

In 2012, the German government proposed the concept of Industry 4.0 [3]. Many companies have started to develop automation technologies, from upper layer software or hardware providers and middle layer companies that store, analyze, manage, and provide solutions, to lower layer companies, so that they can apply these technologies in their factories [4]. In addition, a smarter integrated sensing and control system based on existing automation technologies has begun to evolve as part of the development of a highly automated or even fully automated production model [5].

In modern automation technology, development is mainly based on a variety of algorithms and robot arm control systems [6]. The robotic arm has the advantages of high precision, fast-moving speed, and versatile motion. In advanced applications, extended sensing modules, such as vision modules, distance sensors, or force sensors, can be integrated with the robotic arm to give human sense to robotic systems [7].

Robot systems in general have the ability to “see” things through various vision systems [8]. These help robots to detect and locate objects, which speeds up the technical process. In addition, they improve the processing accuracy and expand the range of applications for automated systems. The type of data obtained can be approximately divided into a 2D vision system (RGB/Mono Camera system), which receives planar images [9], and a 3D vision system (RGB-D Camera system), which receives depth images in the form of vision modules [10]. These two systems have their own advantages and disadvantages and suitable application scenarios. In the field of industrial manufacturing automation, the 2D vision system is commonly used to meet the needs of high precision, ease of installation, and use.

The robotic arm with a 2D vision camera is the most commonly used setup in today’s industrial automation applications. Pick and place [11] and random bin picking [12] are among the most frequent applications that use an industrial camera with a manipulator. Several open-source and commercial toolboxes, such as OpenCV [13] and Halcon [14], are already available for use in vision systems. However, regardless of how mature the software support is, a lot of hardware is still needed with human intervention to complete the process. Camera calibration is needed before operation and is applied to various image processing algorithms so that important parameters, such as focal length, center of image, intrinsic parameter, extrinsic parameter, and lens distortion [15,16], can be learnt. Furthermore, camera calibration is a challenging and time-consuming process for an operator unfamiliar with the characteristics of the camera on the production line. Camera calibration is a major issue that is not easily solved in the automation industry and complicates the introduction of production line automation. Traditional vision-based methods [17,18,19,20] require 3D fixtures corresponding to a reference coordinate system to calibrate a robot. These methods are time-consuming, inconvenient, and may not be feasible in some applications.

A camera installed in the working environment or mounted on a robotic arm can be categorized as an eye-to-hand (camera-in-hand) calibration or stand-alone calibration [21]. The purpose of the hand–eye correction is similar to that of the robot arm end-point tool calibration (TCP calibration), which obtains a convergence homogeneous matrix between the robot end-effector and the tool end-point [22]. However, unlike TCP calibration, it can be corrected by using tools to touch fixed points in different positions. To obtain a hand–eye conversion matrix, the visual systems use different methods, such as the parametrization of a stochastic mode [23] and dual-quaternion parameterization [24], since the actual image center cannot be used. For self-calibration methods, the camera is rigidly linked to the robot end-effector [25]. A vision-based measurement device and a posture measuring device have been used in a system that captures robot position data to model manipulator stiffness [26] and estimate kinematic parameters [27,28,29]. The optimization technique is based on the end-effector’s measured positions. However, these methods require offline calibration, which is a limitation. In such systems, accurate camera model calibration and robot kinematics model calibration are required for accurate positioning. The camera calibration procedure required to achieve high accuracy is, therefore, time-consuming and expensive [30].

Positioning of the manufactured object is an important factor in industrial arm applications. If the object is not correctly positioned, it may cause assembly failure or destroy the object. Consequently, the accuracy of object positioning often indirectly influences the processing accuracy of the automated system. Although numerous studies have been conducted to define object positioning accurately based on vision systems [31,32,33], no system has been found with an offline programing platform to perform AOI inspection on a production line. Table 1 shows a comparison between the system we propose here and to existing vision-based position correction systems in terms of performance. The proposed system expedites the development of a vision-based object position correction module for a robot-guided inspection system. This allows the robot-guided inspection system to complete AOI tasks automatically in a production line regardless of the object’s position. In the proposed system, the AOI targets can be automatically mapped onto a new object for the inspection task, whereas existing vision systems have been developed to locate the object’s position but not for the production line. To operate these systems, the operator needs to be skillful, and integration with the other system is a tedious process. Furthermore, user-defined robot target positions cannot be updated for inspection if there is a change in the object’s position, which makes it more challenging to perform tasks on the production line. The proposed position correction system is capable of self-calibration and can update the object position and AOI targets automatically in the production line.

Here, we propose a novel approach to automate manufacturing systems for various applications in order to solve the object position error encountered on the production line. We developed an automated position correction module to locate an object’s position and adjust the robot pose and position in relation to the detected error values on displacement or rotation. The proposed position correction module is based on an automatic hand–eye calibration and the PnP algorithm. The automatic hand–eye calibration was performed using a calibration board to reduce manual error, whereas the PnP algorithm calculates the object position error using artificial marker images. The position correction module identifies the object’s current position and then measures and adjusts the robot work points for a defined task. This developed module was integrated with the autonomous robot-guided inspection system to build a smart system to perform AOI tasks on the production line. The robot-guided inspection system based on the offline programming (OLP) platform was developed by integrating a 2D/3D vision module [34]. In addition, the position correction module maps the defined AOI target positions to a new object unless they are changed. The effectiveness and robustness of the proposed system was indicated by conducting two tests and comparing captured images with sets of standard images. This innovative system minimizes human effort and time consumption to expedite the AOI setup process in the production line, thereby increasing productivity.

The remainder of this paper is organized as follows: in Section 2, we give an overview of the position correction system integration with robot-guided inspection architecture; in Section 3, we provide an overview of the position correction system and introduce the proposed method; in Section 4, we detail the integration of the position correction module with the OLP platform and report the system performance; in Section 5, we report the conclusions of the proposed system.

## 2. System Overview

**Figure 1:**
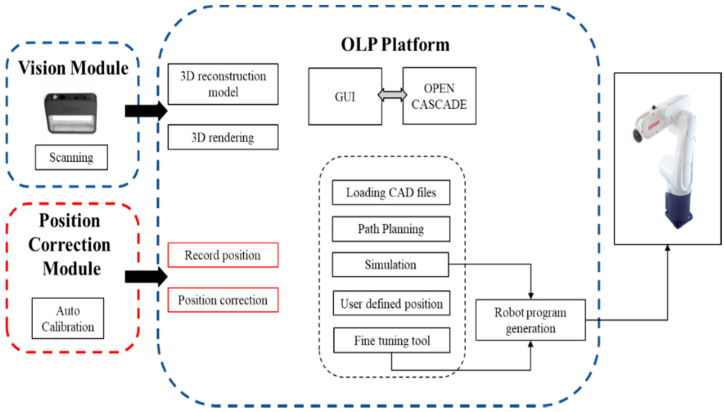
Overview of the position correction module integration with the robot-guided inspection system architecture.

The robot-guided inspection system was designed and developed with the vision module by Amit et al. [34]. The robot-guided inspection system, shown in the blue dashed boxes in Figure 1, consists of an OLP platform and vision module. The OLP platform was designed and developed using OCC open source libraries to generate a robot trajectory for 3D scanning and to define AOI target positions using CAD information [35]. The robot-guided inspection efficiently performs AOI planning tasks using only scanned data and does not require a physical object or an industrial manipulator. This developed system can also be used in different production lines based on robot-guided inspection. However, the developed system is not comprehensive enough to be used in an assembly line to perform reliable AOI tasks. Therefore, the robot-guided inspection system was integrated with the position correction module (red dashed boxes in Figure 1) to resolve issues related to object displacement or rotation errors in a production line for AOI tasks. Figure 1 presents a complete overview of the proposed system architecture, which includes the OLP platform, vision module, and position correction module. In this study, the primary objective of the position correction system was to calculate the rotation and translation of the new object over the production line using artificial markers on the object. Moreover, the proposed system was developed to minimize the complexity of hand–eye calibration and position correction within the production line. In addition, we aimed for the user of the integrated autonomous robot-guided system to not have to define the AOI target positions unless they are changed. This would not only save time and effort, but increase productivity. The proposed position correction system consisted of a simple hand–eye calibration method for the development of the position correction method.

**Figure 2:**
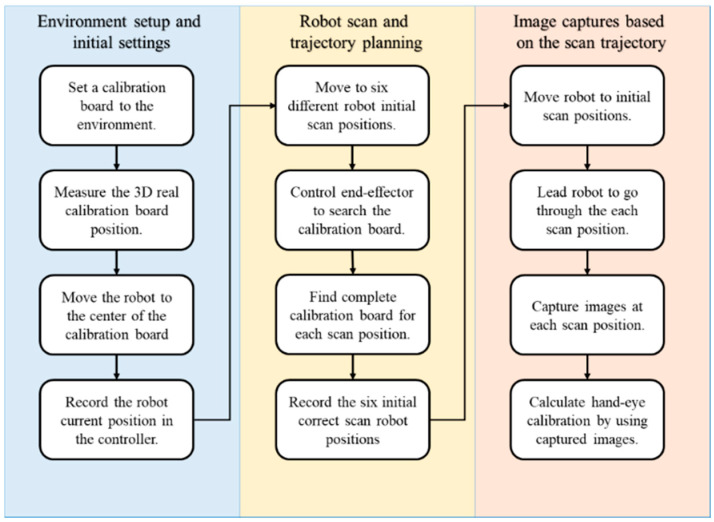
Flowchart of the hand–eye calibration methodology.

The flowchart shown in Figure 2 explains the automatic hand–eye calibration method, which is part of the position correction system. The calibration method was divided into three main stages: “environment setup and initial settings”, “robot scan and trajectory planning”, and “image capture based on the scan trajectory”. In the environment setup and initialization phase, the user must prepare the environment for calibration by measuring the position of the calibration board in the workspace, helping the arm see the calibration board and providing other simple basic settings. The robot scan and trajectory planning stage recorded the optimal end-effector position, while capturing the calibration board image at each position during the image captures based on the scan trajectory stage. The environment setup and initial settings is the only part of the system that requires manual operation (Figure 2). The proposed calibration method was implemented to initiate the position correction module to measure and compensate for the position error before defining the AOI target positions on new objects.

**Figure 3:**
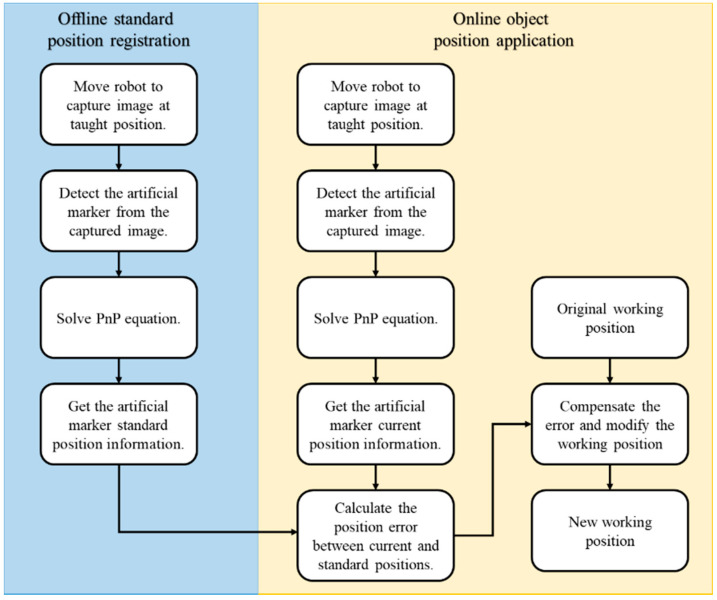
Flowchart of the object position correction methodology.

The flowchart of the proposed object correction methodology is presented in Figure 3 and is divided into the offline registration process and the real-time online object positioning. In the offline process, the user can specify the robot’s artificial marker detection position, design the marker pattern, and work points. In the online process, the system takes a picture of the artificial marker at the specific position based on the user’s offline settings. Subsequently, the system identifies the current object position and autonomously measures and adjusts the robot work points for the AOI inspection task. The position correction system is then integrated with the autonomous robot-guided optical inspection system to demonstrate the performance of the proposed system.

## 3. Overview of the Position Correction System

**Figure 4:**
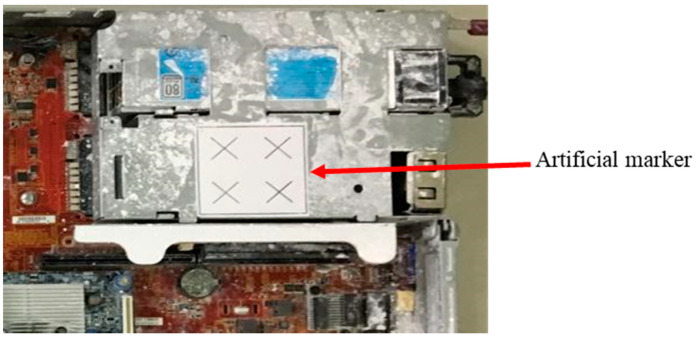
Artificial marker attached to the object.

In this study, an image-based object position correction system was designed and developed. Object positioning has always been a key component of the automated manufacturing process. The proposed position correction system was developed based on a calibration and a position correction methodology. The calibrated camera, with a specific artificial marker on the object for PnP image processing, identifies and defines the position [36,37], as shown in Figure 4. A transformation matrix *T* is then obtained from the marker coordinate (*P_marker_*) to the camera coordinate (*P_Camera_*). The displacement error of the work point is determined by the difference between the object before and after the transformation matrix. The system must then compensate for the position error before defining the AOI target positions on the new object. The system simulation results were evaluated before being integrated with the robot-guided inspection system. This system assists the OLP platform with performing robot-guided AOI applications to automatically inspect misplaced components of manufactured objects on a production line.

### 3.1. Automatic Robot Hand–Eye Calibration Methodology

**Figure 5:**
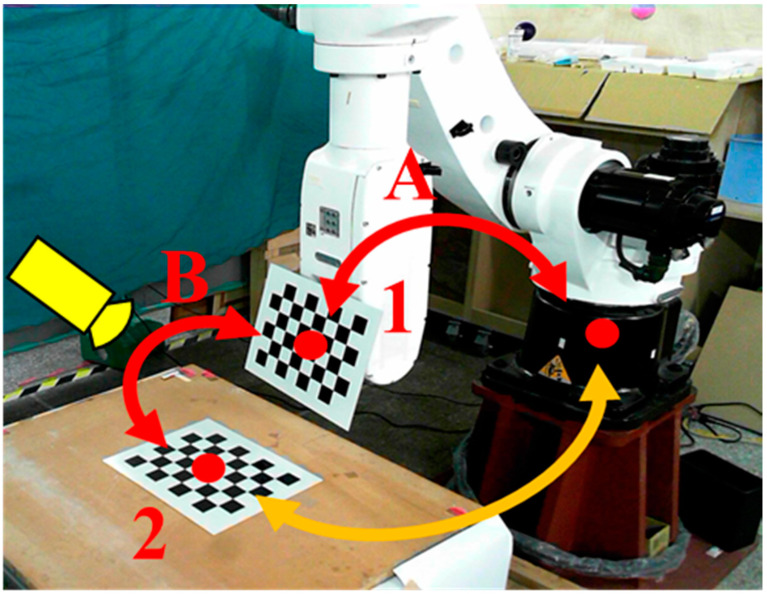
The relationship between robot and two calibration boards.

We evaluated the performance of the proposed automatic hand–eye calibration process shown in Figure 2. The first step of this process was to measure the position of the calibration board in the work space. The calibration board (an 8 × 6 chess board) was fixed on the robot arm and moved closer to the second calibration board on the table [38,39], as shown in Figure 5. We then adjusted the robot arm and camera position so that both calibration boards were visible simultaneously. Two transformation matrices, *A* and *B*, were used to calculate the relationship between the calibration boards. Transformation matrix *A* was obtained using the robot arm controller to record the end-effector coordinates. Transformation matrix *B* was calculated by solving the camera image PnP equations. The transformation matrix between the robot arm and calibration board 2 was calculated after determining the relationship between *A* and *B*, as shown in Figure 5.

**Figure 6:**
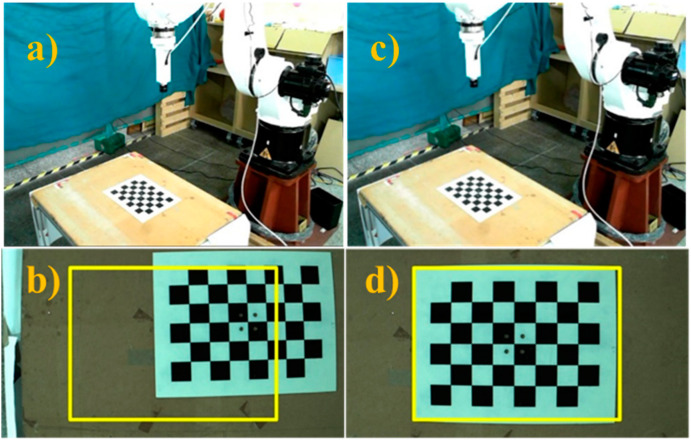
Initial steps of the calibration process. (**a**) Initial robot arm position, (**b**) image captured w.r.t. initial position, (**c**) robot arm position after being manually adjusted, (**d**) image captured after manual adjustment.

**Figure 7:**
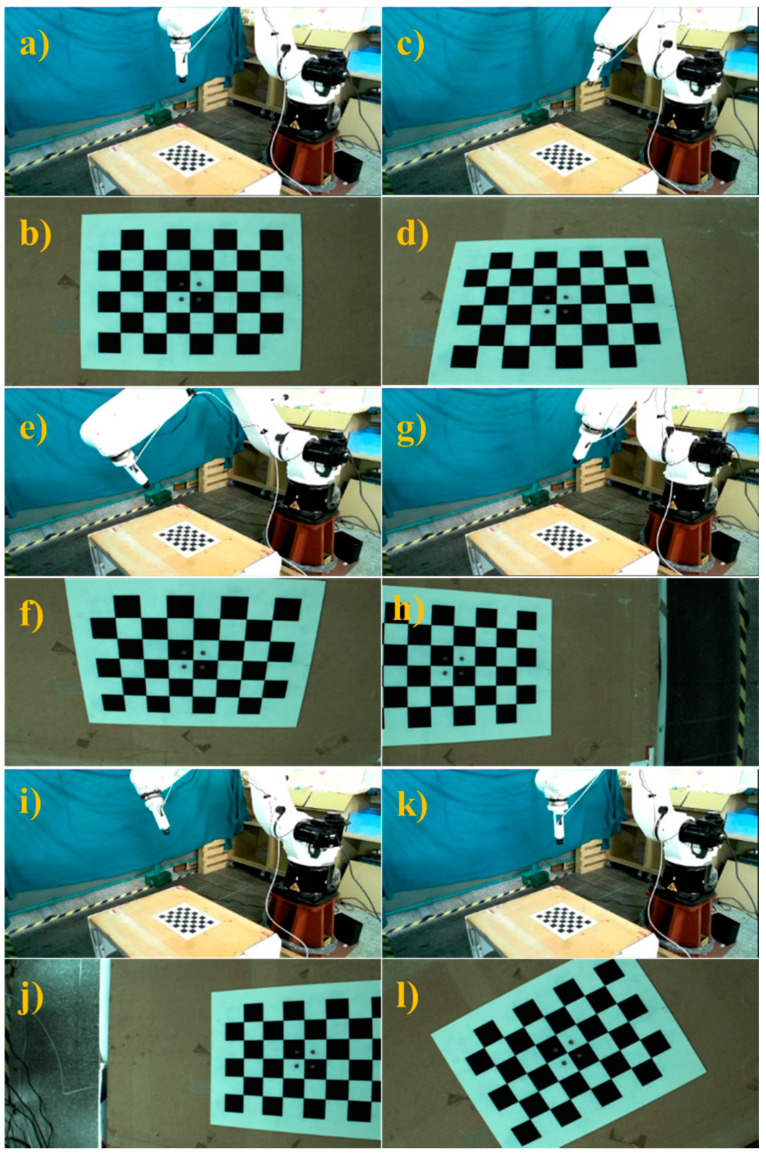
Six different robot positions for automatic hand–eye calibration. (**a**) 1st robot position, (**b**) image w.r.t. 1st position, (**c**) 2nd robot position, (**d**) image w.r.t. 2nd position, (**e**) 3rd robot position, (**f**) image w.r.t. 3rd position, (**g**) 4th robot position, (**h**) image w.r.t. 4th position, (**i**) 5th robot position, (**j**) image w.r.t. 5th position, (**k**) 6th robot position, (**l**) image w.r.t. 6th position.

Once the transformation matrix was obtained, the initial position of the robot was adjusted to perform automatic hand–eye calibration, as shown in Figure 6. The proposed system enabled the robot to follow the hand–eye calibration trajectory and take pictures of the calibration board at different robot positions, as shown in the Figure 7. Table 2 presents each position of the robot end-effector relative to the robot arm’s initial position and the hand–eye calibration results are shown in Table 3. Using these results, the conversion matrix between the robot arm end-effector and the camera connector was calculated and compared with the conversion matrix derived from the original connector design. The proposed calibration method had an error on the *z*-axis of around 2 mm and a greater error of nearly 9 mm on the *x*-axis. The results obtained from the hand–eye correction were sufficiently similar to the designed connector results. Further analysis was performed to identify potential causes of calibration error in order to make further improvements. During this process, errors may have occurred at the 3D printed connector, and the connector center to place the camera was difficult to estimate. In addition, the hand–eye calibration itself is a complex process, and the proposed approach uses the simple AX = ZB closed conversion relationship for inference, so there is a possibility of error. The calibration results obtained were used in the proposed position correction methodology to compute and compensate for the position error on a new object in a production line.

### 3.2. Object Position Correction Methodolgy

After obtaining the calibration results, the user must set the checkpoint position (*P_Check_*) for the robot arm with the camera in the initial “template login phase” to capture the full artificial marker image. The robot arm moves to the (*P_Check_*) position and uses the camera to detect the artificial marker and solve the PnP image problem. Therefore, the transformation matrix *T* of the artificial marker coordinate (*P_marker_*) and camera coordinate (*P_Camera_*) was obtained using Equation (1).

(1) $$P_{Camera}=\left[T\right]P_{Marker}$$

where *T* is a standard position (*T_S_*) and is used as a standard sample to verify and measure the change in object position.

Once the standard position has been obtained, it undergoes the “error compensation phase”, which compensates for the object’s position error during various manufacturing applications. In practice, there are several different work points for various processes of AOI tasks, machining, and assembly applications. The positions of these work points are recorded and are collectively known as *P*. If the object shifts during the process, the robot arm moves to the checkpoint position (*P_Check_*) to detect and resolve the image PnP problem and obtain a new marker position (*P_marker_*). This will calculate a new transformation matrix *T_N_* between the new marker coordinate *P_marker_* and camera coordinate *P_Camera_*.

The offset transformation matrix *T_D_* shown in Equation (2) shows the displacement and rotation of the manual markers and is calculated using the standard transformation matrix *T_S_* and the new transformation matrix *T_N_*.

(2) $$P_{Camera}=\left[T_{N}\right]\;P_{Marker2}=\left[T_{S}\right]\;P_{Marker1}\;P_{Marker2}={\left[T_{N}\right]}^{-1}\left[T_{S}\right]\;P_{Marker1}=\left[T_{D}\right]\;P_{Marker1}\;\left[T_{D}\right]={\left[T_{N}\right]}^{-1}\left[T_{S}\right]$$

The rotation and translation error for defining new work points, *P_New_*_,_ were calculated using the offset transformation matrix, artificial marker center point offset (*S*), and the robot arm’s original coordinates.

Following this, the work point *P* is translated back to the origin of the robot arm coordinate system with the artificial marker center point, as shown in Equation (3):

(3) $$P_{New}=\left[\begin{array}{cc} O & -S\\ O & 1 \end{array}\right]P$$

Work point *P* is then rotated with reference to the artificial marker rotation:

(4) $$P_{New}=\left[\begin{array}{cc} R_{3\times 3} & 0\\ O & 1 \end{array}\right]\left[\begin{array}{cc} O & -S\\ O & 1 \end{array}\right]P$$

Work point *P* is translated back to the original artificial coordinate, as shown in Equation (5):

(5) $$P_{New}=\left[\begin{array}{cc} O & S\\ O & 1 \end{array}\right]\left[\begin{array}{cc} R_{3\times 3} & 0\\ O & 1 \end{array}\right]\left[\begin{array}{cc} O & -S\\ O & 1 \end{array}\right]P$$

Using Equation (6), a new work point (*P_New_*) is defined for the AOI task after error compensation:

(6) $$P_{New}=\left[\begin{array}{cc} O & t_{3\times 1}\\ O & 1 \end{array}\right]\left[\begin{array}{cc} O & S\\ O & 1 \end{array}\right]\left[\begin{array}{cc} R_{3\times 3} & 0\\ O & 1 \end{array}\right]\left[\begin{array}{cc} O & -S\\ O & 1 \end{array}\right]P$$

In summary, translation and rotation error are computed once the object positioning system recognizes and evaluates the artificial marker in the actual application. Based on the proposed approach, this position correction system successfully adjusts and generates new work point coordinates for AOI inspection tasks.

**Figure 8:**
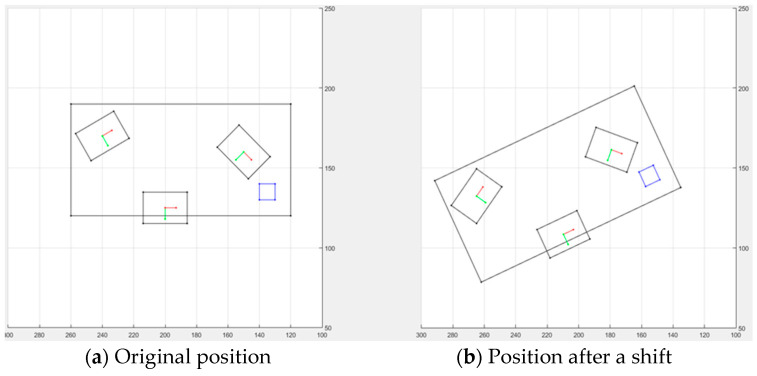
Simulation results of the position correction system. The large black rectangle represents the entire object (computer server board); the blue rectangle is the artificial marker; the three black intermediate size rectangles represent the AOI capture position of the camera; the red and green lines represent the camera’s plane coordinates used to capture the AOI images and the camera coordinate axis, respectively.

The schematic diagram shown in Figure 8 was generated using MATLAB software and was based on the proposed position correction system. In the simulation, the computer server board (object) was moved to an unknown position, after which the position correction system identified the AOI camera target point based on the artificial marker. The performance of the position correction system was high for the AOI target point and object position, regardless of the error caused by the hardware device. The system successfully found the position error and compensated for it to calculate the robot’s new AOI target point, irrespective of the object position. Thus, the object positioning correction system effectively utilized the automatic hand–eye calibration method and simple artificial markers to detect the object’s current position prior to any shifting. Section 4 discusses the integration and implementation of the proposed method in the AOI application, as well as the experimental results.

## 4. Integration of Position Correction Module with OLP Platform

**Figure 9:**
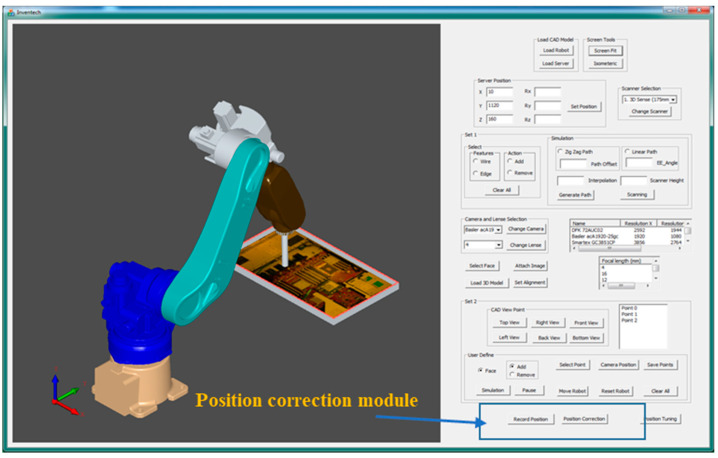
Robot-guided AOI inspection system.

The position correction system we developed was integrated with the autonomous robot-guided optical inspection system to build a smart system for the production line. Prior to performing position correction, a path for real-time scanning and target positions for AOI tasks was generated and visualized graphically in the OLP platform, as shown in Figure 9 [34]. Furthermore, the generated robot program was sent to the HIWIN-620 industrial robot to capture AOI images, which were compared to virtual images. However, the developed system was still unable to reliably perform AOI tasks in a production line. Therefore, the robot-guided inspection system was integrated with the position correction module to resolve the issues related to object displacement or rotation errors in a production line for AOI tasks.

**Figure 10:**
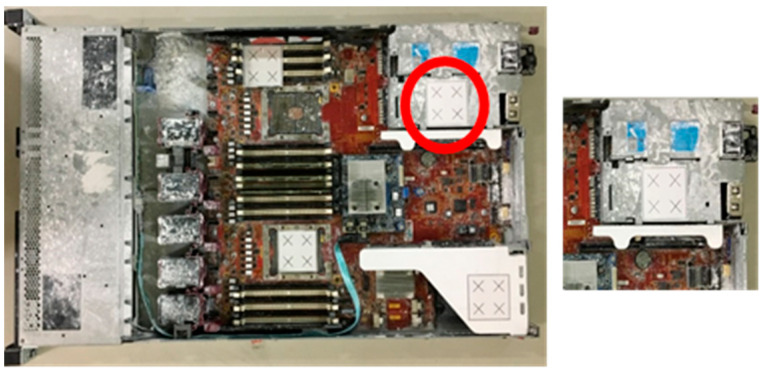
Object positioning system’s target object with artificial markers. The red circle shows the artificial marker (square containing four crosses) used for positioning. The remaining three markers were used to analyze positional errors after camera shooting.

Here, we report and discuss experiments performed using the proposed position correction module for robot-guided inspection. On a production line, the robot arm employs the object position correction module to perform an AOI operation autonomously. To execute an AOI inspection, the robot arm gathers photos of the target object from various angles. If the object shifts, the proposed system detects this and adjusts the robot’s AOI image shooting position. Once positional changes are made, the system captures the defined AOI target images. The object used in this experiment was a large computer server with four artificial markers, as shown in Figure 10.

**Figure 11:**
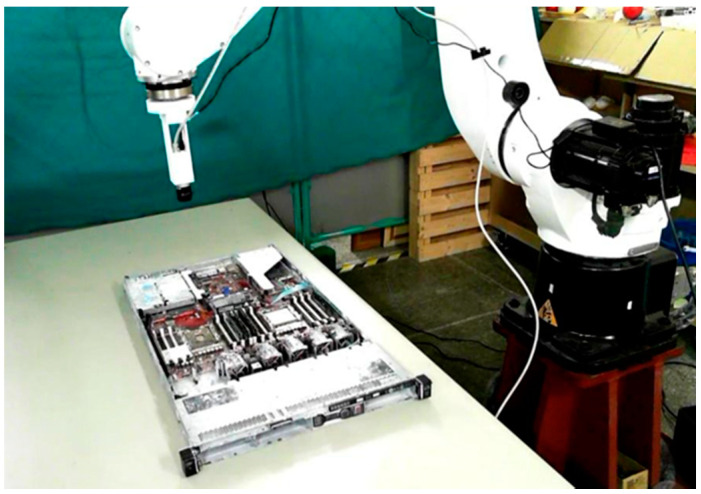
Camera shooting pose at the positioning artificial marker.

**Figure 12:**
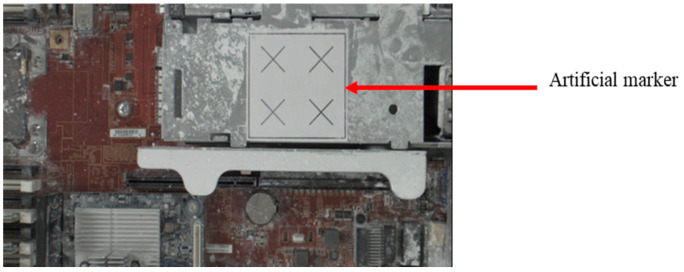
Sample image of the positioning marker w.r.t. the robot pose shown in Figure 11.

During the system execution process, we first manually guided the robot arm to shoot the positioning marker image, as shown in Figure 11. We obtained the sample image of the positioning marker as a reference for the position correction system, as shown in Figure 12, and recorded the positioning marker position and its transformation matrix relative to the camera.

**Figure 13:**
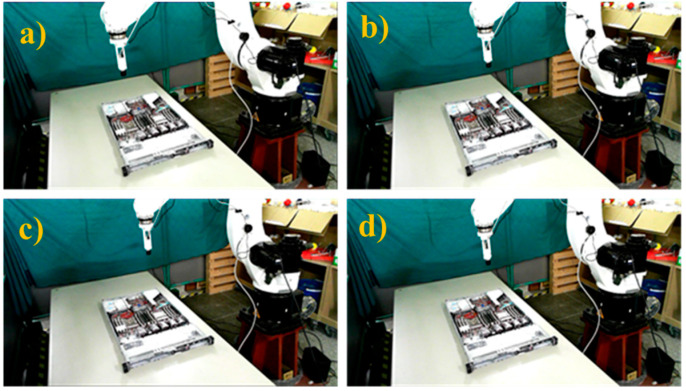
Four different robot poses to capture standard AOI targets images before manual displacement or rotation. Robot pose at the (**a**) 1st, (**b**) 2nd, (**c**) 3rd, and (**d**) 4th AOI target positions.

**Figure 14:**
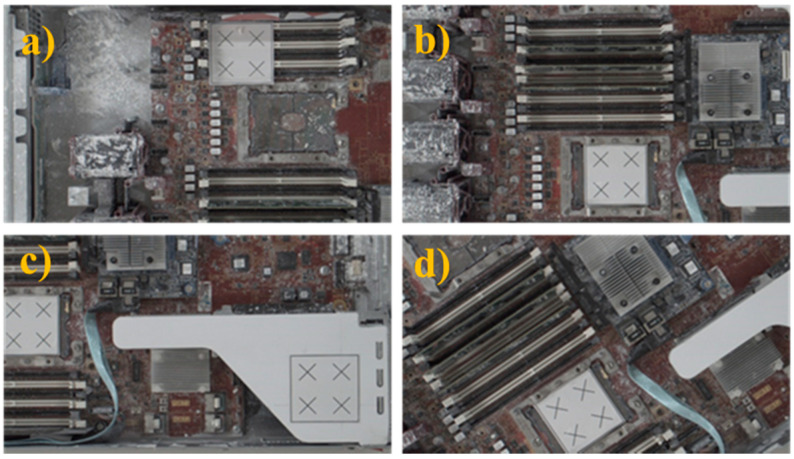
Standard AOI images captured at the four target positions. (**a**) Standard image captured w.r.t Figure 13a, (**b**) Standard image captured w.r.t Figure 13b, (**c**) Standard image captured w.r.t Figure 13c, (**d**) Standard image captured w.r.t Figure 13d.

Once the sample image of the positioning marker was captured, the robot’s target positions were selected for the AOI inspection task before any displacement or rotation. In the experiment, four different robot shooting positions were selected at different heights and angles, as shown in Figure 13, and the images captured at these positions are shown in Figure 14. These were the target points used to perform the position correction procedure.

**Figure 15:**
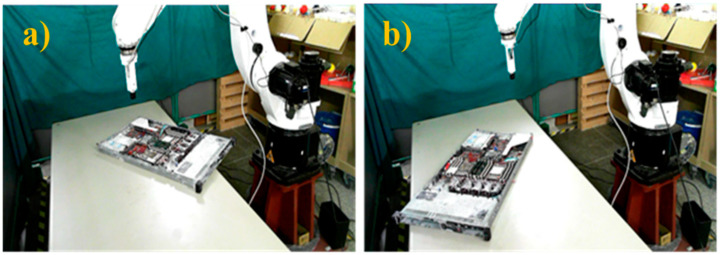
Two different object positions after random manual displacement and rotation to test the object correction system. (**a**) The first and (**b**) second random positions of the object.

**Figure 16:**
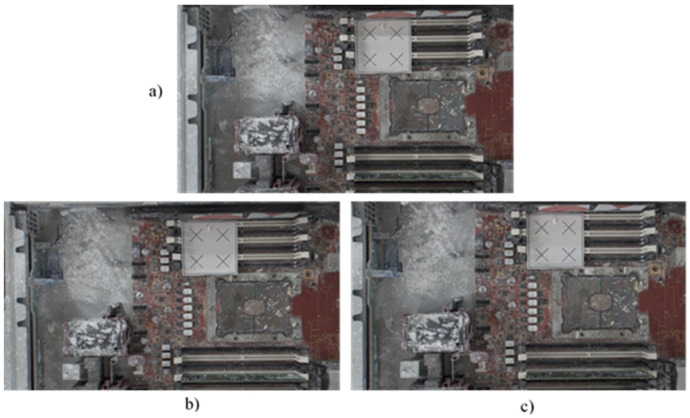
Position correction system test results for two random positions. Standard image without displacement or rotation corresponding to (**a**) Figure 14a, and new images after (**b**) the first random position shown in Figure 15a and (**c**) the second random position shown in Figure 15b.

**Figure 17:**
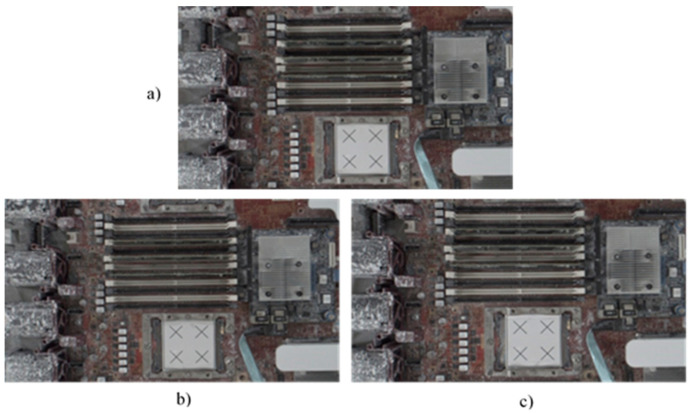
Position correction system test results for two random positions. Standard image without displacement or rotation corresponding to (**a**) Figure 14b, and new images after (**b**) the first random position shown in Figure 15a and (**c**) the second random position shown in Figure 15b.

**Figure 18:**
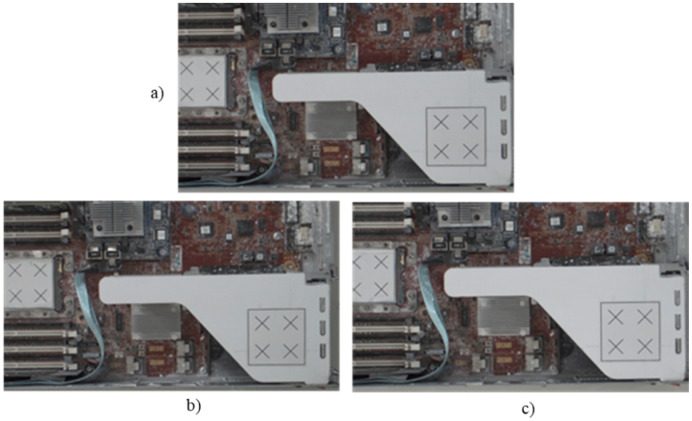
Position correction system test results for two random positions. Standard image without displacement or rotation shown in (**a**) Figure 14c, and new images after (**b**) the first random position shown in Figure 15a and (**c**) the second random position shown in Figure 15b.

**Figure 19:**
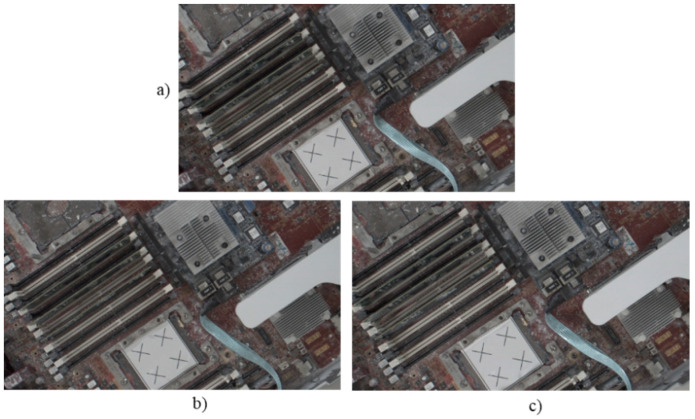
Position correction system test results for two random positions. Standard image without displacement or rotation shown in (**a**) Figure 14d, and new images after (**b**) the first random position shown in Figure 15a and (**c**) the second random position shown in Figure 15b.

Following this preparatory procedure, the system already had all of the parameters and specification data required to perform object image repositioning. Figure 14 shows the original standard image without displacement. Two experiments with random manual displacement were conducted to evaluate the performance of the proposed position correction module, as shown in Figure 15. Once the manual displacement was carried out, the proposed position correction modules calculated the new AOI position to capture images, as shown in Figure 16, Figure 17, Figure 18 and Figure 19. These were then compared with the standard images shown in Figure 14.

**Figure 20:**
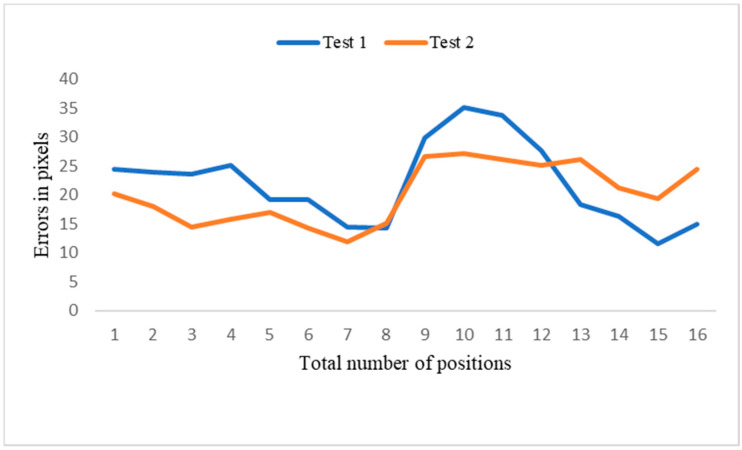
The distance error between test 1 and test 2.

**Figure 21:**
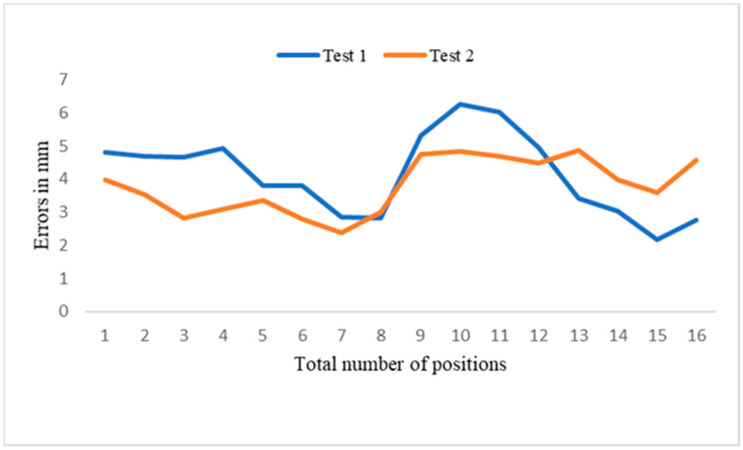
Distance error between test 1 and test 2.

Two experiments were conducted to measure the performance of the position correction system. In test 1, the object was moved by 49.1 mm, with a final average residual error of 5.15 mm, and the proposed system compensated for 91.5% of the position error. In test 2, the object was moved 50.6 mm, with a final average residual error of 3.80 mm, and the proposed system compensated for 92.5% of the position error. New images were captured and compared with the standard images for the two random object positions. The captured images differed slightly from the standard image. These results demonstrate that the proposed system efficiently compensates for most of the error caused by object displacement. Table 4 presents the error analysis of the object position correction system, in which new images have a distance error (norm) of 10–40 pixels compared to the standard image (Figure 20), with an average of 21.09 pixels. The errors measured in pixels were then converted to mm (Table 5). The distance error was between 6 and 2 mm (Figure 21) and the mean error was 3.97 mm. The proposed method’s efficiency could be improved by accurately positioning the camera and replacing the 3D print camera holder, as well as minimizing calibration error, which may improve system accuracy by up to 95.3%. The developed system resolved the issues associated with the object translation or rotation error in the production line. The robot-guided inspection system with position correction module enhanced the ability to perform user-defined AOI tasks autonomously on any production line.

## 5. Conclusions

In this study, a novel position correction module was developed and integrated with an autonomous robot-guided optical inspection system to perform AOI tasks on production lines and correct for object displacement and rotation. The robot-guided system assisted the user to select the AOI targets and capture target images in the virtual environment. Real-time images were captured using the industrial manipulator for the corresponding positions. However, this system was still not reliable enough to be used in an assembly line to perform AOI tasks. Therefore, the robot-guided inspection system was integrated with a position correction module to resolve object displacement or rotation error issues in a production line for AOI tasks. The position correction system calculates and compensates for the position error of the new object on the production line using artificial markers. We performed two tests to evaluate the effectiveness of the proposed position correction module. The proposed system had a mean error of 3.97 mm or 21.09 pixels. These results indicated that the robot-guided system with a position correction module was capable of performing AOI tasks on a production line. In addition, the user of the integrated autonomous robot-guided system is not required to define the AOI target on a new object position unless they are changed. This not only saves time and effort, but also increases productivity. The integration of the position correction module with the robot-guided system led to the development of a smart system, which enables inspection tasks on the production line without prior knowledge of the object’s position. However, the proposed method is currently limited to using artificial markers and the simple AX = ZB closed conversion for hand–eye calibration.

## Figures and Tables

**Table 1:** Comparison between existing vision-based position correction systems and the proposed system.

Features	Vision-Based Systems [31,32,33]	Proposed System
Self-calibration	Yes	Yes
Errors and failures	High	Low
Online position correction in a production line	No	Yes
Setup complexity with OLP	High	Low
Auto correct robot targets	No	Yes

**Table 2:** The six different positions of the robot end-effector relative to the initial position.

Position	*X*	*Y*	*Z*	*R_X_(α)*	*R_Y_(β)*	*R_Z_(γ)*
A	0	0	0	0	0	0
B	0	+30 cm	+10 cm	+30°	0	0
C	0	–30 cm	–10 cm	–30°	0	0
D	–30 cm	0	0	0	+30°	0
E	+30 cm	0	0	0	–30°	0
F	0	0	0	0	0	+30°

**Table 3:** Hand–eye calibration results and error.

Position	Connector Design Size	Hand–Eye Calibration Results	Error
d*X* (mm)	0	−8.559	8.559
d*Y* (mm)	0	−0.227	0.227
d*Z* (mm)	190	189.387	1.613
*Rx* (degrees)	0	−0.079	0.079
*Ry* (degrees)	0	−0.868	0.868
*Rz* (degrees)	0	−0.874	0.874

**Table 4:** Error analysis of the object position correction system (measured in pixels).

**Error** **(in Pixel)**	**First Displacement**			**Second Displacement**		
	***X***	***Y***	***Norm***	***X***	***Y***	***Norm***
**AOI position 1**						
P1	1.11	24.39	24.41	−7.29	−18.92	20.28
P2	0.26	23.89	23.89	−4.26	−17.44	17.95
P3	0.32	23.69	23.69	−4.52	−13.72	14.45
P4	1.46	25.02	25.06	−7.50	−13.87	15.77
**AOI position 2**						
P1	0.44	19.16	19.16	−14.16	−9.02	17.04
P2	−3.52	18.91	19.23	−10.49	−9.60	14.22
P3	−3.50	14.05	14.48	−10.59	−5.62	11.99
P4	0.87	14.31	14.34	−14.40	−4.59	15.11
**AOI position 3**						
P1	−25.55	15.93	29.83	0.64	−26.58	26.59
P2	−31.64	15.09	35.06	0.01	−27.12	27.12
P3	−32.66	8.48	33.74	0.01	−26.13	26.13
P4	−26.29	8.76	27.71	0.56	−25.08	25.08
**AOI position 4**						
P1	16.36	8.34	18.36	−25.67	4.65	26.08
P2	12.36	10.61	16.28	−21.25	1.20	21.28
P3	10.16	5.75	11.67	−18.65	5.03	19.31
P4	14.52	3.36	14.90	−22.78	8.97	24.49

**Table 5:** Error analysis of object position correction system (in mm).

**Error** **(in mm)**	**First Displacement**			**Second Displacement**		
	***X***	***Y***	***Norm***	***X***	***Y***	***Norm***
**AOI position 1**						
P1	0.22	4.79	4.80	−1.43	−3.72	3.98
P2	0.05	4.69	4.69	−0.83	−3.43	3.53
P3	0.06	4.65	4.65	−0.89	−2.70	2.84
P4	0.29	4.92	4.92	−1.47	−2.73	3.10
**AOI position 2**						
P1	0.09	3.79	3.79	−2.86	−1.79	3.37
P2	−0.70	3.74	3.80	−2.08	−1.90	2.81
P3	−0.69	2.78	2.86	−2.10	−1.11	2.37
P4	0.17	2.83	2.84	−2.85	−0.91	2.99
**AOI position 3**						
P1	−4.57	2.75	5.33	0.11	−4.76	4.76
P2	−5.65	2.70	6.26	0.00	−4.85	4.85
P3	−5.84	1.52	6.03	0.00	−4.68	4.68
P4	−4.70	1.57	4.95	0.10	−4.49	4.49
**AOI position 4**						
P1	3.05	1.55	3.43	−4.79	0.87	4.87
P2	2.31	1.98	3.04	−3.96	0.22	3.97
P3	1.90	1.07	2.18	−3.48	0.94	3.60
P4	2.71	0.63	2.78	−4.25	1.67	4.57
